# Too much is too much: Influence of former stress levels on food craving and weight gain during the COVID-19 period

**DOI:** 10.1371/journal.pone.0277856

**Published:** 2023-04-27

**Authors:** Rachel Granger, Hans P. Kubis

**Affiliations:** 1 Centre for Health Economics and Medicines Evaluation, School of Medical and Health Sciences, Bangor University, Bangor, United Kingdom; 2 School of Human and Behavioural Sciences, College of Human Sciences, Bangor University, Bangor, United Kingdom; University of Petra (UOP), JORDAN

## Abstract

The COVID-19 pandemic and associated social restrictions had an extensive effect on peoples’ lives. Increased rates of weight gain were widely reported, as were declines in the general populations’ mental health, including increases in perceived stress. This study investigated whether higher perceived levels of stress during the pandemic were associated with greater levels of weight gain, and whether poor prior levels of mental health were a factor in higher levels of both stress and weight gain during the pandemic. Underlying changes in eating behaviours and dietary consumption were also investigated. During January-February 2021, UK adults (n = 179) completed a self-report online questionnaire to measure perceived levels of stress and changes (current versus pre-COVID-19 restrictions) in weight, eating behaviours, dietary consumption, and physical activity. Participants also reported on how COVID-19 had impacted their lives and their level of mental health prior to the pandemic. Participants with higher levels of stress were significantly more likely to report weight gain and twice as likely to report increased food cravings and comfort food consumption (OR = 2.3 and 1.9–2.5, respectively). Participants reporting an increase in food cravings were 6–11 times more likely to snack and to have increased consumption of high sugar or processed foods (OR = 6.3, 11.2 and 6.3, respectively). Females reported a far greater number of COVID-19 enforced lifestyle changes and both being female and having poor mental health prior to the pandemic were significant predictors of higher stress and weight gain during the pandemic. Although COVID-19 and the pandemic restrictions were unprecedented, this study suggests that understanding and addressing the disparity of higher perceived stress in females and individuals’ previous levels of mental health, as well as the key role of food cravings, is key for successfully addressing the continuing societal issue of weight gain and obesity.

## Introduction

After the declaration of the SARS-CoV-2 coronavirus (COVID-19) pandemic in March 2020 [1], restrictions were put in place in many countries around the world, to help curb the spread of the virus [2]. This included the government-imposed restrictions that came into place in the UK on 23rd March 2020 [3]. These initial COVID-19 restrictions caused sudden and radical changes in habits and lifestyles, including self-isolation for individuals more vulnerable to the virus, physical distancing, transfer to home-based working and education, closure of indoor community spaces and sports facilities, use of personal protective equipment (PPE) in work, and job furlough in many sectors [4]. The subsequent lockdowns in Autumn 2020 and Winter 2020–21 (timings varied between countries in the UK) had a significant impact on the UK population [4].

Although COVID-19 restrictions were implemented to improve the health outcome of the pandemic, the high level of hospitalisations and deaths in the UK [5] caused concern both over personal health and the health of loved ones [6]. These concerns, combined with financial insecurity, due to increased job uncertainty and losses [7], and reduced social interactions were linked to a considerable increase on the levels of psychological stress reported by general populations in the UK [6, 8] and around the world [9–12].

Since the start of the pandemic there was also an overall reported increase in food intake and weight gain both in the UK and other countries [13–18]. Studies in the first few months of the pandemic reported weight gains in 22–50% of general adult populations [15–18], with follow up studies reporting a substantial subset of people who maintained or reported further weight gain [17, 19]. These increases in weight gain were associated with specific dietary behaviours, including increased snacking [14] and greater consumption of processed foods, including high sugar and high fat/energy foods [20–22]. This increased weight gain was also often associated with increased levels of food cravings or reductions in food craving control [17, 19, 20, 22, 23].

Prior to COVID-19, obesity was already considered to be a global epidemic [24–26]. In addition, people with obesity are likely to suffer more serious health consequences from COVID-19 infection [27, 28]. Obesity is a complex multifactorial condition, who’s development is influenced by genetic and behavioural factors, but also by wider socioeconomic and environmental influences of health, which are often linked to higher levels of stress [29]. The level of stress experienced by an individual will often be greater when there is a high perceived level of uncertainty, unpredictability, lack of information and lack of control [30]; all factors that were present during the COVID-19 pandemic.

Prior to the pandemic, long-term chronic stress already had a significant positive association with weight gain [31–34]. Several studies during the pandemic have already reported a link between increased stress and: weight gain [17, 19, 20, 22], changes in eating behaviours [35] or changes in dietary consumption [36].

Food cravings are defined an intense desire to consume a particular food or food type, often in the absence of hunger [37]. Higher levels of food cravings are associated with individuals who have higher BMI/obesity [38] or disordered eating patterns, such as bulimia [20, 39]. Higher levels of food cravings or loss of craving control have also been linked to reduced weight loss and early drop-out rates in dietary interventions [39]. Food cravings are generally associated with foods that are high in sugar, fat/energy and are often highly processed [20, 23, 40]. These foods are also often referred to as comfort foods, as their consumption in excessive amounts is associated with an attempt to sooth emotional upset due to stress, anxiety, depression, loneliness, or anger [41, 42]. Excessive consumption of comfort food is also a main driver of weight gain [43].

Some of the main barriers to weight loss and weight maintenance that have been identified in recent studies are mental distress and binge eating (or comfort eating) [44], negative thoughts or moods [45] and traumatic life events [46]. Recent reviews on the role of stress in weight gain has suggested that rather than being viewed as primarily drivers of weight gain, cravings and comfort eating should be considered as mediators of the effect of stress on dietary intake and weight gain [24, 32].

The aim of this study was to investigate whether higher levels of stress reported by the general UK population during the pandemic were linked to higher levels of weight gain, and whether these changes were directly associated with increased food cravings and comfort eating. Secondly, we aimed to investigate whether individuals’ level of mental health prior to the pandemic were linked to higher levels of stress and weight gain during the pandemic. In addition, we addressed whether specific lifestyle changes caused by COVID-19 and the UK imposed restrictions were linked to higher levels of stress. We hypothesized that individuals with higher perceived stress would report greater weight gain during the pandemic, with people who had poor mental health before COVID showing the strongest effects. Furthermore, we hypothesized that the weight gain and perceived stress were strongly linked with increased food cravings and comfort food consumption.

## Materials and methods

### Participants

The study used a convenience sample and the eligibility criteria was that participants were aged 18 or over and had been resident in the UK since the beginning of COVID-19 restrictions in March 2020. Participants were recruited through open adverts on social media and through internal advertising in Bangor University School of Human and Behavioural Sciences. To achieve adequate power for testing a minimum of 92 participants were required, based on a two tailed priori power analysis of 25% effect size, 0.8 power, 0.05 error (calculated using GPower Software). Effect size used was based on previously reported increases in food intake and weight gain when under high levels of stress, both prior to and during COVID-19 restrictions [15, 17]. The study protocol was performed in accordance with the declaration of Helsinki and was approved by Bangor University School of Human and Behavioural Sciences Ethics Committee (ethics approval number M012021).

### Measurements

Data was collected using a bespoke online survey that was administered via Qualtrics (Qualtrics Software Company Provo, UT, USA). The survey was completed anonymously, with no data collected that could identify participants. The survey was designed to be easy to answer and completed in as short a time as possible, to ensure minimal drop out of participants. To enable this, where appropriate, questions were asked about categorical changes (i.e., increased, stayed the same or reduced) rather than absolute measures. As well as simplifying the questionnaire, the use of categorical measures, rather than absolute measures, enabled the reporting of overall trends while avoiding inaccuracy problems with self-report measures.

At the start of the survey participants were provided with a brief overview of the study aims and the participant inclusion criteria (over 18 and based in the UK since the beginning of COVID-19 restriction). For participants to take part in the study they were required to click on a button of “I meet the inclusion criteria and consent to take part in the study”. After providing informed consent participants reported on the following information:

#### Demographics and weight change

Participants reported on the demographic measures on age, gender, main current occupation, country of the UK based, height and weight (with BMI being calculated by the researcher). Participants were asked to report how their weight had changed since the beginning of the COVID-19 restrictions within the following categories: lost more than 3kg/6 pounds, lost up to 3kg/6 pounds; weight remained stable, gained up to 3kg/6 pounds, gained between 3kg/6 pounds and 6kg/a stone, gained more than 6kg/a stone.

#### Eating behaviours

Participants were asked to report whether their general hunger, food cravings, number of meals consumed, snacking in the daytime and snacking in the evening had decreased, increased or stayed the same in the past 3 months, compared to prior to COVID-19 restrictions. Participants also reported if they believed any of the following reasons had increased their dietary consumption: being at home, boredom, stress anxiety or low moods, feeling tired or lacking in energy, The behaviour categories were based on categories reported by other COVID-19 studies [20, 47].

#### Dietary consumption

Participants were asked to report if their consumption of any of the following categories had decreased, increased or remained the same in the past 3 months, compared to before the start of the COVID-19 restrictions: fruit and veg; high fibre foods (such as wholemeal food, wholegrains and beans), red or processed meat (including bacon, ham, sausages; white meat (including fish), dairy products, vegetarian/vegan meat or dairy alternatives, processed foods (including ready meals or takeaways), home cooked food, tinned or frozen food; high sugar foods (such as cakes, biscuits, chocolates and sweets; high fat foods (such as savoury snacks, crisps or nuts), coffee or tea, fruit juices or smoothies, fizzy drinks (including diet drinks), alcohol, water. The range of dietary categories was based on dietary categories reported in other COVID-19 studies [15–17].

#### Physical activity

To ensure that any reported changes in weight were due to changes in dietary consumption rather than changes in physical activity, participants were asked if their level of vigorous/moderate exercise, walking and sedentary time had decreased, increased or stayed the same in the past 3-months compared to before COVID-19 restrictions.

#### Stress

As part of the survey participants completed the Perceived Stress Scale PSS-10 [48] to evaluate general levels of stress (Cronbach’s α = 0.87, for this study). In addition, participants also reported whether prior to COVID restrictions they: had occasional stresses but generally could cope well, had some level of stress anxiety or low mood but not diagnosed, had previously been diagnosed with stress anxiety or depression, or preferred not to say.

#### Impact of COVID-19 on lifestyle

Participants were asked to report on ways in which ways COVID-19 and the associated imposed restrictions had impacted their lifestyle and food availability. Participants were asked if the restrictions had impacted them in any of the following ways: continued to work outside the home but had to use personal protective equipment (PPE), moved to work from home, experienced job uncertainly, experienced job loss, had confirmed COVID-19 infection, had to self- isolate due to possible COVID-19 infection, had to self-isolate due to underlying health issues, had additional caring responsibilities including school-aged children or other dependents, had less access to fresh or healthy food, had less money to spend on food, had less time to prepare healthy food.

### Data analysis

Descriptive statistics (mean ±SD) were used to characterise survey respondents and summarise their changes in weight gain, eating behaviours, dietary consumption and physical activity. Eating behaviours and dietary consumption were re-categorised in to an “increased” or “no increase” categories (which included the “stayed the same” and “decreased” categories). This was as this study was specifically interested in the likelihood of increased food cravings and comfort foods being linked to stress and weight gain.

Although the PSS-10 questionnaire gives a continuous score outcome, to enable us to carry out logistic analysis with the categorical variables reported for changes in weight, dietary consumption and exercise, participants were recategorized into a higher or lower stress group, with categorization based on the mean PSS-10 score of trial participants (19.3±6.9). Participants with a PSS score of 19.3 or lower were allocated to the lower stress category and participants with a PSS score of 19.4 or higher were allocated to the higher stress category.

Ordinal regression analyses were used to identify factors linked to a greater likelihood of reporting weight gain. Pearson’s chi-square analyses were used to investigate a significant difference in likelihood in response between the higher and lower stress groups to: weight gain, increases in eating behaviours, increases in dietary consumption, and changes in physical activity. Binary regression analysis was used to identify factors linked to a greater likelihood of belonging to the higher stress group. Odds ratios were also calculated for all chi-square analyses, to allow direct comparison of results with the binary and ordinal regression analyses. All statistical analyses were carried out using the Statistical Package for Social Sciences (IBM SPSS Statistics for Windows V.27.0. Armonk, USA). A 95% confidence interval (95% CI) was selected, and a significance level of *p* < 0.05 was applied to all statistical analyses.

## Results

### Descriptive statistics including impact of COVID-19 restrictions

In total, responses were collected from 220 participants between 6th January until 2nd February 2021. After removing duplicates and incomplete questionnaires, 179 participants’ data sets remained. There was no significant demographic difference between survey completers and non-completers. The full survey dataset is available in S1 Dataset.

Most survey respondents were female (75%) and aged between 18–55 (85%). Two thirds of respondents were based in Wales (66%) and a third (34%) in England. The mean BMI of respondents was 26.10(±5.46) kg/m^2^.

Many respondents reported that their main current occupation involved working from home, with a higher proportion of females being based at home (F = 40%:M = 27%) and more male respondents continuing to work outside the home (F = 15%:M = 27%). As the questionnaire was advertised within the university, unsurprisingly students made up 25% of respondents, although 8% of responders were in the retired category. Notably, only females reported that caring for children or dependents was their main occupation (5%). The overall level of participants unemployed or furloughed was low (3% and 1%, respectively).

When asked about the impact of COVID-19 restrictions on their lifestyle, a comparable number of women and men reported moved to work from home (F = 50%: M = 46%). A higher proportion of women reported worked outside the home using PPE (F = 35%:M = 25%), having to isolate due to potential COVID-19 infection (F = 25%:M = 16%) or having additional caring responsibilities for children or other dependents (F = 39%:M = 9%). Males reported higher levels of job uncertainty (F = 12%:M = 23%), job loss (F = 5%:M = 7%), having had COVID-19 (F = 4%:M = 7%) and self-isolating due to underlying health issues (F = 4%:M = 11%). The difference in the level of impact of COVID-19 restrictions by gender was notable. When comparing the total number of changes that participants were affected by, 53% of females were affected by 2 or more lifestyle changes compared to 39% of males. The impact of COVID-19 restrictions on food availability was also far more likely to impact females. Females were around twice as likely to report less money to spend on food (F = 16%:M = 9%), less access to healthy food (F = 12%:M = 7%) or less time to cook healthy food (F = 11%:M = 5%). Full demographic statistics for survey participants are available in S1 Table.

### Stress and weight gain

Participants were asked to report their weight change in categories rather than absolute weight changes to avoided inaccuracy problems with self-report measures. There was a notable difference between the genders for reported weight gain, with females almost twice as likely as males to report an overall weight gain since the start of the COVID-19 restrictions (F = 50%: M = 27%). and twice as likely to report the highest level of weight gain of more than 6kg (F = 10%: M = 5%). see Table 1 for all reported weight changes.

**Table 1:** Changes in weight reported since the start of COVID-19 restrictions.

Weight change	Female	Male	Total
	n = (%)	n = (%)	n = (%)
**Gained more than 6kg**	13 (9.6%)	2 (4.5%)	15 (8.4%)
**Gained 3-6kg**	17 (12.6%)	3 (6.8%)	20 (11.2%)
**Gained up to 3kg**	37 (27.4%)	7 (15.9%)	44 (24.6%)
**Weight remained the same**	40 (29.6%)	24 (54.5%)	64 (35.8%)
**Lost up to 3kg**	12 (8.9%)	1 (2.3%)	13 (7.3%)
**Lost more than 3kg**	16 (11.9%)	7 (15.9%)	23 (12.8%)

Ordinal regression analysis showed that the likelihood of the higher stress group reporting a greater level of weight gain was statistically significant, with a medium effect size, χ^2^(5, n = 179) = 14.639, *p* = 0.012, *phi* = 0.286). This confirmed the study’s main hypothesis that participants reporting a higher level of stress during the pandemic were statistically more likely to report a higher level of weight gain. Although the aim this study was not specifically focus on gender differences, due to the large differences in weight changes reported by males and females a Chi-square tests for independence was carried out, which showed that the association between gender and weight gain was statistically significant, with small to medium effect, χ^2^(2, n = 179) = 11.986, *p* = 0.035, *phi* = 0.259).

### Stress, eating behaviours and weight gain

In the survey, participants were asked to report on changes in eating behaviours that are often linked to weight gain, including hunger, number of meals, cravings, and snacking. Overall increases were reported for all eating behaviour categories, apart from number of meals (see Table 2). Although most respondents (53%) reported that their levels of general hunger had remained at pre-COVID-19 levels, over half of respondents reported that their food cravings, snacking in the day and snacking in the evening had increased (52%, 53% and 53%, respectively). Half of respondents also reported that the reason they had eaten more was due to being at home or boredom (52% and 48%, respectively), with 41% reporting eating more due to stress, anxiety or low mood (see Table 3).

**Table 2:** Changes in reported levels of dietary behaviours.

Eating Behaviours	Decreased	Stayed the same	Increased
	n = (%)	n = (%)	n = (%)
**General hunger**	27 (15.2%)	94 (52.8%)	57 (32.0%)
**Food cravings**	18 (10.2%)	66 (37.5%)	92 (52.3%)
**Number of meals**	24 (13.5%)	131 (73.6%)	23 (12.9%)
**Snacking in the day**	31 (17.4%)	52 (29.2%)	95 (53.4%)
**Snacking in the evening**	21 (12.1%)	61 (35.1%)	92 (52.9%)

**Table 3:** Reasons given for increased food consumption.

Reasons for changes	Yes	No
	n = (%)	n = (%)
**Being at home**	93 (52.0%)	86 (48.0%)
**Boredom**	85 (47.5%)	94 (52.5%)
**Stress, anxiety or low mood**	75 (41.9%)	104 (58.1%)
**Tired or lacking in energy**	53 (29.6%)	126 (70.4%)

An ordinal logistic analysis was performed to assess whether increases in the 5 eating behaviours and 4 reasons given for increased eating increased the likelihood of respondents reporting weight gain. The full model was statistically significant, χ^2^(9, n = 179) = 70.877, *p*<0.001), with increased food cravings (p = 0.002, OR = 3.225), increased number of meals (p = 0.012, OR = 2.978), increased snacking in the day (p = 0.012, OR = 2.568) and eating due to stress, anxiety or low mood (*p* = 0.026, *OR* = 2.192) all significant factors linked to weight gain. Although being at home or boredom were given by the participants for the primary reasons for increased food consumption, there was no statistical significance of these factors and actual weight gain. Table 4 shows all coefficients in the ordinal logistic model.

**Table 4:** Coefficients of the predictive model for weight gain.

				95% CI for Exp(B)	
Variables Entered	B	SE	OR	LL	UL
**Increase in General Hunger**	-.183	.3464	.833	.423	1.643
**Increase in Food Cravings**	1.171	.3783	3.225*	1.536	6.768
**Increase in Number of Meals**	1.091	.4337	2.978*	1.273	6.967
**Increases in Snacking in the Day**	.943	.3766	2.568*	1.227	5.372
**Increases in Snacking in the Evening**	.520	.3326	1.682	.876	3.228
**Ate more due to being at home**	-.119	.3738	.888	.427	1.848
**Ate more due to boredom**	-.352	.3327	.703	.366	1.350
**Ate more due to stress**	.785	.3535	2.192*	1.096	4.382
**Ate more due to low energy**	.339	.3479	1.403	.709	2.775

* = *p*<0.05

** = *p*<0.001. n = 179. *SE* = standard error of B. *OR* = odds ratio. *LL* = lower limit; *UL* = upper limit

To determine whether these behaviours were more likely in the higher stress group a chi-square test was carried out. This showed that participants who were in the higher stress category were twice as likely to report increased food cravings *χ*^*2*^ (1, n = 179) = 6.402, *p* = 0.011, *phi* = 0.202;) *OR* = 2.3 and snacking in the day *χ*^*2*^ (1, n = 179) = 4.192, *p* = 0.041, *phi* = 0.165), *OR* = 1.9. Participants in the higher stress category were also more likely to report increased eating due to stress, anxiety or low mood *χ*^*2*^ (1, n = 179) = 25.684, *p*<0.001, *phi* = 0.390), which is as expected, as both measures are reporting on levels of stress. In comparison, there was no statistical significance for higher stress and increased number of meals. In summary, increased food cravings and snacking in the day were the only behaviours correlated with both higher stress and weight gain.

### Stress, dietary consumption and weight gain

Like changes in eating behaviours, there was a polarisation of changes dietary consumption. Over half of respondents maintained their pre-COVID-19 consumptions levels of fruit and veg (55%), high fibre (70%), red/processed meats (52%), white meat/fish (60%), dairy products (71%), vegetarian/vegan alternatives (62%) and tinned/frozen foods (68%). A notable change was seen with home cooked food category, where 50% increased their consumption. The other categories which showed the greatest change in consumption were those that can be categorised as comfort foods. There was comparable split between decreased, increased or remained the same for processed foods (36%, 29% and 30% respectively). Forty percent of participants reported an increase in consumption of high fat/salt foods, and over half (57%) of participants reported an increase in high sugar foods. With the drink categories, the majority respondents reported maintaining their pre-COVID-19 consumption level of fruit juices/smoothies, fizzy drinks and water (59%, 54% and 58%, respectively). In comparison almost half of respondents reported increased consumption of coffee and tea, and alcohol (42% and 46%, respectively). A full summary of the dietary changes for survey participants is available are available in S2 Table.

To determine which dietary categories were linked to weight gain an ordinal logistic regression was carried out. The full model, containing all 16 food and drinks categories, was statistically significant, *χ*^*2*^(16, n = 179) = 75.291, *p*<0.001), although only 4 of the 16 categories made a significant contribution. Increased consumption of high sugar and processed foods were linked to a 2.5–3.2-times likelihood of an increase in weight gain (*p* = 0.005, *OR* = 3.2 and *p* = 0.032, *OR* = 2.5, respectively). However, the inverse effect was seen in the categories of high fibre foods (*p* = 0.034, *OR* = 0.4) and water (*p* = 0.033, *OR* = 0.5), where increased consumption was linked to a greater likelihood of weight loss. Table 5 shows all coefficients in the ordinal logistic model.

**Table 5:** Food and drink coefficients of the predictive model for weight gain.

Category	B	Std. Error	OR	Lower	Upper
**Increase in Fruit and Veg**	.445	.3786	1.561	.743	3.279
**Increase in Fibre**	-1.048	.4941	.351*	.133	.923
**Increase in Red/processed meat**	-.318	.3817	.727	.344	1.537
**Increase in white meat, fish and eggs**	-.637	.3901	.529	.246	1.136
**Increase in dairy**	.850	.4590	2.339	.951	5.751
** Increase in vegetarian and vegan**	-.639	.3829	.528	.249	1.118
** Increase in processed/take aways/ready meals**	.917	.4274	2.501*	1.082	5.780
**Increase in Home cooked**	-.631	.3431	.532	.272	1.043
**Increased in Tinned/Frozen**	-.332	.4978	.717	.270	1.903
**Increase in High Sugar foods**	1.147	.4102	3.148*	1.409	7.035
**Increase in high salt foods**	.426	.4200	1.531	.672	3.487
**Increase in Coffee or Tea**	.420	.3256	1.521	.804	2.880
**Increase in Juice/smoothies**	.163	.4800	1.177	.460	3.016
**Increase in Fizzy drinks**	.114	.3647	1.120	.548	2.290
**Increase in Alcohol**	.534	.3290	1.706	.895	3.251
**Increase in Water**	-.788	.3695	.455*	.220	.938

* = *p*<0.05

** = *p*<0.001. n = 179. *SE* = standard error of B. *OR* = odds ratio. *LL* = lower limit; *UL* = upper limit

A chi-square analysis was carried out to determine whether the dietary categories linked to weight change were also linked to higher levels of stress. This showed that participants in the higher stress group were twice as likely to have increased their consumption of both high sugar foods *χ*^*2*^(1, n = 179) = 4.001, *p* = 0.045, phi = 0.161), *OR* = 1.9 and processed foods *χ*^*2*^ (1, n = 179) = 6.310, *p* = 0.012, *phi* = 0.201), *OR* = 2.5. In comparison, there was no statistical significance for changes in the consumption of high fibre food or water and higher levels of stress. In summary, increases in high sugar and processed food consumption were the only dietary categories linked both to a higher level of stress and weight gain.

### Relationship between food cravings and comfort eating

To investigate further the relationship between increased food cravings and increased comfort food consumption (which includes the high sugar, high fat/salt and processed food categories), a chi-square analysis was performed. We also investigated if an increase in food cravings was linked to an increase in snacking, as comfort food is often consumed outside mealtimes [49]. Participants who reported increased food cravings were over 11 times more likely to report increased consumption of high sugar foods *χ*^*2*^(1) = 48.008, *p*<0.001, *phi* = 0.534; *OR* = 11.2), and over 6 times more likely to report increased consumption of processed foods, *χ*^*2*^ (1) = 22.920, *p*<0.001, *phi* = 0.374, *OR* = 6.3), although there was no significant link with high fat/salt foods. There was also a statistically significant link between increased cravings and increased snacking in the day (but not snacking in the evening), with participants reporting increased food cravings 6 times more likely to report an increase in snacking during the day, *χ*^*2*^ (1) = 29.181, *p*<0.001, *phi* = 0.419; *OR* = 6.0).

### Physical activity and weight gain

To discount the role of physical activity in the effect of stress on weight gain, participants were also asked to report in their changes in physical activity. Almost half of respondents (47%) reported a reduction in their level of moderate or strenuous activity compared to their pre-COVID-19 levels of activity. In comparison, almost half (43%) of participants reported that they had increased their level of walking compared to pre-COVID-19 level. However, the category with the greatest reported difference, was sedentary behaviour, with 78% or respondents reporting an increase in sedentary compared to their pre-COVID-19 levels. Chi-squared tests for level of activity versus weight change did show a statistical significance for a reduction in moderate/strenuous activity and weight gain, *χ*^*2*^(2, n = 179) = 25.354, *p* = 0.005, *phi* = 0.271), but not for changes in level of walking or sedentary time. Chi-squared tests of level of activity versus stress levels were non-significant *χ*^*2*^(2, n = 179) = 1.40, *p* = 0.50, *phi* = 0.088). Consequently, although a reduction of moderate/strenuous activity increased the likelihood of weight gain, it was not a mediator of the effect of stress on weight gain. A full summary of the physical activity changes for survey participants is available in S2 Table.

**Fig 1:**
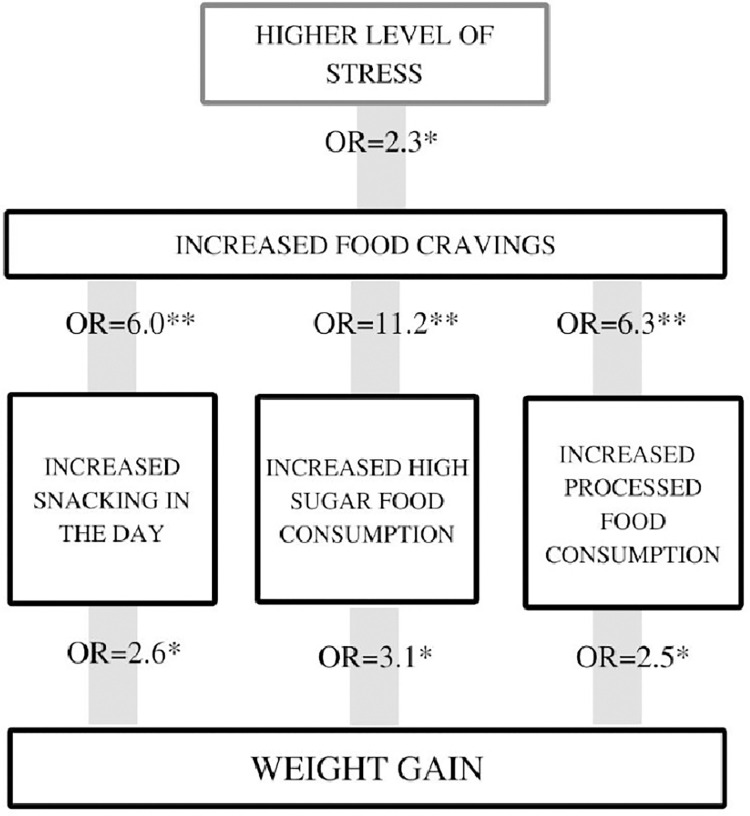
Summary of the key statistical analyses from the study. n = 179; *OR* = odds ratio; * = *p*<0.05; ** = *p*<0.001.

Fig 1 summarises all the significantly statistical findings reported, highlighting the corelation between higher stress, eating behaviours, dietary consumption and weight gain.

### Factors that impacted stress levels during COVID-19

Participants were asked to report both their current levels of perceived stress, using the PSS-10 questionnaire, and their mental health prior to the start of the pandemic. With both categories a higher level of stress was reported by women. The overall PSS-10 score for trial participants was (19.3±6.9). However, the average score for women was 20.4±6.6, compared to an average score of 15.7±6.7 for men. Half of participants (54%) reported that prior to COVID-19 they had generally coped well with stress. 25% reported that they had some level of stress, anxiety or depression prior to COVID-19 restrictions, but had not received a formal diagnosis and 17% reported that they had previously been diagnosed with stress, anxiety or depression. Again, women were far more likely than men to report having been diagnosed with stress, anxiety or depression prior to COVID-19 (F = 19%:M14%) or have had some level of stress anxiety or depression but had not been diagnosed (F = 26%:M = 20%).

As the pandemic caused so many enforced changes to peoples’ everyday life, one of the aims of this study was to see if any of these changes directly increased participants level of perceived stress. As reported in the descriptive statistics, moving to work from home was the highest reported change and was comparable for women and men. There were also notable differences between the genders in the numbers of changes they were affected by and some key categories such as caring from children and other dependents. A binary regression analysis was carried out which included all lifestyle changes, and gender, age and mental health prior to COVID-19. Surprisingly, the analysis did not show a significant link between any of the lifestyle changes and higher levels of perceived stress. However, gender and mental health prior to COVID-19 were both significantly linked to perceived stress during the pandemic χ2(18, n = 179) = 58.95, p<0.001). Participants who had a prior diagnosis of stress, anxiety or depression were almost six times more likely to be in the higher stress category (p = 0.004, OR = 5.7), confirming our hypothesis, with participants who had not been officially diagnosed, but reported some levels of stress, anxiety, or depression over three times more likely to be in the higher stress category (p = 0.004, OR = 3.6). Females were around 4 times more likely to be in the higher stress category than males (*p* = 0.014, *OR* = 3.6), which was independent of their levels of stress prior to COVID-19. Table 6 shows the results for all coefficients of the model.

**Table 6:** Coefficients of the predictive model for high stress (PSS score = 20 or above).

				95% CI	
Variables Entered	B	SE	OR	LL	UL
**Female sex (vs male)**	1.172	.479	3.228*	1.264	8.247
**Age 18–30**	2.112	1.167	8.262	.839	81.312
**Age 31–40**	1.340	1.190	3.821	.371	39.380
**Age 41–55**	.677	1.154	1.967	.205	18.889
**Age 56–70**	.925	1.207	2.522	.237	26.876
**Previously diagnosed with stress/anxiety or depression**	1.752	.579	5.767*	1.853	17.951
**Some level of stress, anxiety or depression, but not diagnosed**	1.298	.448	3.663*	1.523	8.812
**Continuing to work outside the home with PPE**	.692	.441	1.998	.842	4.743
**Moved to work from home**	.289	.404	1.335	.605	2.946
**Job uncertainty.**	1.003	.620	2.728	.808	9.203
**Job loss**	.652	1.030	1.920	.255	14.468
**Have had COVID-19**	-.485	1.077	.616	.075	5.079
**Self-isolated due to potential COVID-19 infection**	.098	.489	1.103	.423	2.878
**Self-isolated due to underlying health issues**	1.034	.883	2.812	.498	15.876
**Caring for children or other dependents**	.562	.463	1.754	.708	4.349
**Restriction on food due to less time to cook**	.841	.684	2.320	.607	8.870
**Restriction on food due to access to food**	1.331	.808	3.784	.777	18.421
**Restriction on food due to less money**	.585	.634	1.796	.518	6.222

* = p<0.05

** = p<0.001. n = 179. SE = standard error of B. OR = odds ratio. LL = lower limit; UL = upper limit

## Discussion

The aim of this study was to assess whether higher levels of perceived stress during COVID-19 restrictions were linked to an increased likelihood of reporting weight gain, and whether increases in food cravings and comfort eating were mediators of this effect. We also investigated whether specific pandemic induced lifestyle changes were linked to a higher level of perceived stress and whether prior levels of mental health were linked to a greater likelihood of perceived stress during the restrictions.

Participants of this study reported an overall gain in weight since the start of the pandemic. which was specifically linked to increases in food cravings and snacking. There was also a clear association between reported weight gain and increased dietary consumption of processed foods, high fat/salt foods, and in particular high sugar foods, with the interaction between behaviours and consumption summarised in Fig 1. We reported that participants who increased snacking or consumption of high sugar and processed foods were 2.5–3.1 times more likely to report weight gain. Participants who reported increased food cravings were 6 times more likely to increase their snacking and processed food consumption and over 11 times more likely to increase their consumption of high sugar foods. These changes were also reported by most participants, with 52% reporting an increase in cravings, 53% an increase in snacking and 57% an increased consumption of high sugar foods. Our results are also consistent with previous studies, which have reported 42–46% increases in cravings, 53–56% increases in snacking and 28–45% increase in consumption of high sugar foods during the pandemic [15, 20, 22, 23], indicating that these are realistic measures of the changes in populations’ dietary habits. This is particularly concerning, as it indicates that around half of these populations have made dietary changes during the pandemic that are strongly associated with weight gain and if continued longer-term with obesity. Our study was carried out 10 months since the start of the pandemic, indicating that these may already be long-term changes in people’s dietary behaviour. Other studies also similar longer-term changes in people’s behaviour [17, 19].

This study also specifically investigated the relationship between stress and weight gain. Our results showed that higher levels of stress were positively associated with weight gain and participants with high levels of stress were twice as likely to report increased food cravings. As discussed in the introduction, higher levels of food cravings have been reported in individuals who have higher BMI/obesity and disordered eating patterns [20, 38, 39] and are likely to be mediators of the effect of stress on dietary intake and weight gain [24, 32]. Like the reported changes in dietary consumption, over half (52%) of participants in this study reported an increase in food cravings. Other published studies have also reported a significant link with stress and weight gain. In a large American study weight-gainers reported higher stress levels and less craving controls during the initial lockdown period than non-weight gainers. For those who continued to weight gain after the lockdowns eased, consumption of high ultra-processed food in conjunction with low craving control and continued levels of stress were the significant drivers [19]. Other studies have reported similar effects. with links between increased stress and weight gain [17, 20, 22], changes in eating behaviours [35] or changes in dietary consumption [36]. These results again indicate that food cravings (or lack of food craving control) are a key factor in understanding and mitigating the effect of stress on weight gain.

This study investigated the link between poor mental health prior to the pandemic and these changes. We reported that those who had a mental health (psychiatric) diagnosis prior to the pandemic were almost six times more likely to be in the higher stress category (OR = 5.7). In addition, participants who had not been officially diagnosed, but reported some prior levels of stress, anxiety, or depression were also three times more likely to be in the higher stress category (OR = 3.6). Other studies have also reported prior mental health, stress and weight gain. Participants with a diagnosis of psychiatric illness or obesity prior to the pandemic were more likely to report declines in weight gain protective behaviours [50]. Having a diagnosis of depression pre-pandemic was reported to be associated with weight gain during the pandemic (OR 1.54) [51]. Further study reported that anxiety and/or depression were the strongest predictors of weight gain in the general population [52]. In addition, a study specifically focused on weight gain in overweight and obese patients during lockdown reported that although stress and low depression were weight gain predictors for all participants, for those with a psychiatric diagnosis prior to COVID-19 binge eating was additional behavioural predictor of weight gain [53]. These findings all indicate that poor mental health was a significant driver of weight gain during the pandemic, with individuals with a mental health diagnosis more likely to gain weight and display disordered eating patterns, both of which have been independently linked to higher levels of stress and food cravings [20, 39]

Whilst this study was not specifically designed to investigate differences by gender, our results reported significant differences between women and men. Women reported higher levels of weight gain (F = 50%: M = 27%) and were twice as likely to have reported the highest weight gain category (F = 10%: M = 5%). Women were more likely to have a mental health diagnosis prior to COVID-19 (F = 19%;M = 14%) or have low mental health but not diagnosed (F = 26%;M = 20%). In addition, during the study they were also over 3 times more likely to be in the higher stress category than males (O*R* = 3.6), which was independent of their levels of mental health prior to COVID-19. Other published studies have also highlighted higher stress levels during COVID-19 in women than in men [10–12], although not all studies have found a difference by gender [51]. This suggests that it may be gender associated factors rather than being female that may be the key to the level of stress reported.

To investigate the potential causes of increased stress during the pandemic participants of this study were asked to report on ways in which ways COVID-19 and the associated imposed restrictions had impacted their lives. Similar to stress levels, we found that there was a disproportionate impact on female respondents. Higher levels of working outside the home with PPE were reported by women, suggesting that they were more likely to work in areas in close proximity to other people, with a likely higher associated risk of infection. Despite the level of COVID-19 infection being reported as higher for men, women reported higher levels of having to isolate due to potential infection, indicating that they may have been in environments with a higher risk of infection, either through work or possibly due to caring roles. This is likely to have led to increased disruption and uncertainty both for work and their personal lives. Although these changes would be expected to cause an increase in stress, none of the individual lifestyle changes showed a statistically significant link with higher levels of stress in this study. It should be considered that specific combinations of factors or the overall number of factors may have an been required to have an impact on stress. However, unfortunately this study was not designed to capture that level of complexity of analysis. In comparison, other studies have shown that specific lifestyle changes, for example caring responsibilities, were associated with increased stress and weight gain. A study reported that in people caring for children during the pandemic stress was associated with both higher levels of emotional eating and depression, particularly for those concerned about weight gain prior to the pandemic [54]. Another study reported that having children at home was a significant factor of reported weight gain [52]. Both these studies and our results highlighted the disparity between the percentage of men and women in caring roles, including additional responsibilities due to the pandemic-imposed changes such as home-schooling. They also indicate that it may have been both the number as well as the type of additional roles that women tock on that impacted their level of stress. As the unequal burden on women looks to continue in the UK with the current cost of living crisis, acknowledging the role of stress in weight gain and focusing on effective strategies for stress management are likely to be key for women for effective weight loss and maintenance.

Limitations to this study are acknowledged. Although self-report surveys are useful to investigate overall trends, we acknowledge that they may not be a true reflection of actual behaviours and are often prone to bias, as they are based on responders’ perceptions of their behaviour [55]. To minimise this, questions on impact of COVID-19 restrictions and changes in eating behaviours and dietary consumption were based on previous surveys in other populations which enabled the comparison of results across similar populations. In addition, a range of general questions for eating behaviours and dietary consumption were included (rather than just asking about behaviours of interest) to limit the focus on what might perceived as negative behaviour. The study only asked about levels of change, rather than absolute measurements, so all measurements are based on comparable rather than quantitative levels of change. We also acknowledge body measurements (weight and height) collected by self-reporting are prone to inaccuracy and variation (both due to individual participants’ techniques and different measurement tools being used). However, by using self-report method the number of participants was not restricted to those who could be measured by the researcher nor by the pandemic restrictions in place at the time. Self-reported body composition measurements were also used by many published studies on weight changes during COVID-19, so this study is comparable to previously published work.

It is acknowledged that this is a relatively small survey, with most respondents (85%) being within in the 18–55 age range. We believe, however, that the study results are valid, as the survey achieved adequate power (as reported in the materials and methods section). In addition, the results are in line with other similar larger published studies cited in this paper. With respondents being mainly young-middle aged adults, we recognise that this population is not fully representative of the UK adult population. However, as many of the COVID-19 imposed restrictions (such moved to work from home, caring for dependents etc) were more likely to impact the working age population, we believe that with this study sample enabled a realistic evaluation the impact of the pandemic restrictions on people’s lives.

The primary aim of this study was to investigate the relationship between higher stress and weight gain for a wide range of factors. To enable us to do this using logistic analyses we categorised all factors. However, it is accepted that by using categorical rather than continuous measures for perceived stress scores we may have limited the ability to track smaller changes in lifestyle factors on stress levels, particularly as former studies indicate that females routinely report higher levels of stress [10–12]. We also accept that by categorising other factors, such as age, we may not have been able to identify more subtle overall changes that continuous data would have allowed.

Perhaps the most important limitation of this work that it is a correlational study, so although we are proposing that higher stress leads to weight gain, it needs to be considered that weight gain could also be the driver of stress. We acknowledge that there are complex interactions between stress and weight gain, with different responses shown in females and males [56]. However, we proposed that by focusing on the role of food cravings this provides a stronger indication that the main direction of influence is stress, which drives food cravings, which in turn causes weight gain.

## Conclusions

In summary, the results from this study corroborate previous research, that higher levels of stress are significantly linked to increased weight gain. The study also highlights significant differences in perceived stress and weight gain during the pandemic by gender and the role in mental health prior to the pandemic in weight gain. Further work is necessary to understand how stress reduction strategies could be incorporated into weight loss/maintenance approaches, including a greater awareness of individuals’ stress levels and history of mental health when developing weight loss and maintenance strategies. A more gendered approach to weight loss may also be required, either to take into consideration differences in perceived stress levels between females and males or to determine if the role of stress in weight gain is a gendered factor.

The study also reiterates the key role of food cravings in the link between stress and weight gain. Food cravings were linked to a 3 times increased likelihood of reporting weight gain, and to 6–11 times increased likelihood of snacking and consumption of high sugar and-processed foods. This strongly suggests that re-focusing weight loss methods on the ability to control cravings, possibly by using stress reduction techniques, will be a more effective approach to than the current focus on reduction of the downstream behaviours of increased snacking, and consumption of processed and high sugar foods.

## Supporting information

S1 Dataset Provided in SPSS format. (ZIP)

S1 Table Descriptive statistics: Demographics, reported changes due to COVID-19 and levels of stress. (DOCX)

S2 Table Reported changes: In dietary behaviours, dietary consumption and physical activity. (DOCX)
