# Supplementary material for: Too much is too much: Influence of former stress levels on food craving and weight gain during the COVID-19 period
Source: PLoS One. 2023 Apr 27;18(4):e0277856. doi: 10.1371/journal.pone.0277856 (PMC10138263; doi:10.1371/journal.pone.0277856)
Supplement: S1 Table — (DOCX) [file pone.0277856.s002.docx]

**S2 Table Descriptive statistics:** demographics, and reported changes due to COVID-19 and levels of stress

|  | **Female**  **n= (%)** | **Male**  **n= (%)** | **Total**  **n= (%)** |
| --- | --- | --- | --- |
| **Total** | 135 (75.4%) | 44 (24.6%) | 179 (100%) |
| **Age** |  |  |  |
| 18-30 | 42 (31.1%) | 17 (38.6%) | 59 (33.0%) |
| 31-40 | 24 (17.8%) | 6 (13.6%) | 30 (16.8%) |
| 41-55 | 50 (37.0%) | 13 (29.5%) | 63 (35.2%) |
| 56-69 | 14 (10.4%) | 6 (13.6%) | 20 (11.2%) |
| 70 or over | 5 (3.7%) | 2 (4.5%) | 7 (3.9%) |
| **Area based** |  |  |  |
| Wales | 90 (66.7%) | 28 (63.6%) | 118 (65.9%) |
| Scotland | 1 (0.7%) | 0 (0.0%) | 1 (0.6%) |
| N Ireland | 0 (0.0%) | 0 (0.0%) | 0 (0.0%) |
| England | 44 (32.6%) | 16 (36.4%) | 60 (33.5%) |
| **BMI kg/m^2^** | 26.3 (±5.7) | 25.5 (±4.6) | 26.1 (±5.5) |
| **Main Current Occupation** |  |  |  |
| Working outside the home | 20 (14.8%) | 12 (27.3%) | 32 (17.9%) |
| Working at home | 54 (40.0%) | 12 (27.3%) | 66 (36.9%) |
| Furloughed | 1 (0.7%) | 1 (2.3%) | 2 (1.1%) |
| Student | 31 (23.0%) | 13 (29.5%) | 44 (24.6%) |
| Retired | 10 (7.4%) | 4 (9.1%) | 14 (7.8%) |
| Caring for children/dependents | 7 (5.2%) | 0 (0.0%) | 7 (3.9%) |
| Unemployed | 3 (2.2%) | 2 (4.5%) | 5 (2.8%) |
| Other | 9 (6.7%) | 0 (0.0%) | 9 (5.0%) |
| **Lifestyle changes** |  |  |  |
| Working outside the home with PPE | 47 (34.8%) | 11 (25.0%) | 58 (32.4%) |
| Moved to home working | 67 (49.6%) | 20 (45.5%) | 87 (48.6%) |
| Job uncertainty | 16 (11.9%) | 10 (22.7%) | 26 (14.5%) |
| Job loss | 7 (5.2%) | 3 (6.8%) | 10 (5.6%) |
| Have had COVID-19 | 6 (4.4%) | 3 (6.8%) | 9 (5.0%) |
| Self-isolated due to potential COVID-19 infection | 33 (24.4%) | 7 (15.9%) | 40 (22.3%) |
| Self-isolated due to underlying health issues | 5 (3.7%) | 5 (11.4%) | 10 (5.6%) |
| Additional caring for children or other dependents | 52 (38.5%) | 4 (9.1%) | 56 (31.1%) |
| **Number of lifestyle changes affected by** |  |  |  |
| Not affected by any changes | 13 (9.6%) | 8 (18.2%) | 21 (11.7%) |
| Affected by 1 change | 50 (37.0%) | 19 (43.2%) | 69 (38.5%) |
| Affected by 2 changes | 43 (31.9%) | 10 (22.7%) | 53 (29.6%) |
| Affected by 3 changes | 20 (14.8%) | 5 (11.4%) | 25 (14.0%) |
| Affected by 4 or more changes | 9 (6.6%) | 2 (4.6%) | 11 (6.1%) |
| **Covid restrictions and food availability** |  |  |  |
| Less access to fresh healthy food | 16 (11.9%) | 3 (6.8%) | 19 (10.6%) |
| Less money to spend on food | 22 (16.3%) | 4 (9.1%) | 26 (14.5%) |
| Less time to cook healthy food | 15 (11.1%) | 2 (4.5%) | 17 (9.5%) |
| **Number of food availability factors affected by** |  |  |  |
| Not affected by any factors | 93 (68.9%) | 35 (79.5%) | 128 (71.5%) |
| Affected by 1 factor | 33 (24.4%) | 9 (20.5%) | 42 (23.5%) |
| Affected by 2 or more factors | 9 (6.7%) | 0 (0.0%) | 9 (5.0%) |
| **PSS score (score range of 0 to 40)** | 20.4 (±6.6) | 15.7 (±6.7) | 19.3 (±6.9) |
| High stress category (score of 20 or more) | 80 (59.3%) | 15 (34.1%) | 95 (53.1%) |
| Low stress category (store of 19 or less) | 55 (40.7%) | 29 (65.9%) | 84 (46.9%) |
| **Previous level of Stress** |  |  |  |
| Previously diagnosed with stress/anxiety or depression | 25 (18.5%) | 6 (13.6%) | 31 (17.3%) |
| Some level of stress, anxiety or depression, but not diagnosed | 35 (25.9%) | 9 (20.4%) | 44 (24.6%) |
| Occasional stresses, but generally coped well | 69 (51.1%) | 28 (63.6%) | 97 (54.2%) |
| Prefer not to answer | 6 (4.4%) | 1(2.3%) | 7 (3.9%) |
