# Supplementary material for: Too much is too much: Influence of former stress levels on food craving and weight gain during the COVID-19 period
Source: PLoS One. 2023 Apr 27;18(4):e0277856. doi: 10.1371/journal.pone.0277856 (PMC10138263; doi:10.1371/journal.pone.0277856)
Supplement: S2 Table — (DOCX) [file pone.0277856.s003.docx]

**S3 Table:** Reported Changes in Dietary Behaviours, Dietary Consumption and Physical Activity

| **Eating habits** | **Decreased**  **n= (%)** | **Stayed the same**  **n= (%)** | **Increased**  **n= (%)** |
| --- | --- | --- | --- |
| **Eating Behaviours** |  |  |  |
| General hunger | 27 (15.2%) | 94 (52.8%) | 57 (32.0%) |
| Food cravings | 18 (10.2%) | 66 (37.5%) | 92 (52.3%) |
| Number of meals | 24 (13.5%) | 131 (73.6%) | 23 (12.9%) |
| Snacking in the day | 31 (17.4%) | 52 (29.2%) | 95 (53.4%) |
| Snacking in the evening | 21 (12.1%) | 61 (35.1%) | 92 (52.9%) |
| **Dietary Consumption** |  |  |  |
| Fruit and veg | 21 (11.7%) | 99 (55.3%) | 59 (33.0%) |
| High Fibre | 27 (15.1%) | 125 (69.8%) | 27 (15.1%) |
| Red and processed meat | 41 (24.3%) | 87 (51.5%) | 41 (24.3%) |
| White meat, fish & eggs | 24 (13.8%) | 105 (60.3%) | 45 (25.9%) |
| Dairy products | 28 (16.0%) | 124 (70.9%) | 23 (13.1%) |
| Vegetarian/vegan | 27 (15.4%) | 108 (61.7%) | 40 (22.9%) |
| Processed | 63 (35.6%) | 62 (35.0%) | 52 (29.4%) |
| Home cooked | 13 (7.3%) | 75 (42.4%) | 89 (50.3%) |
| Tinned or frozen | 34 (19.0%) | 122 (68.2%) | 23 (12.8%) |
| High sugar | 30 (16.9%) | 47 (26.4%) | 101 (56.7%) |
| High salt/fat | 39 (22.3%) | 66 (37.7%) | 70 (40.0%) |
| Coffee or tea | 17 (9.6%) | 86 (48.3%) | 75 (42.1%) |
| Fruit juice | 48 (27.7%) | 102 (59.0%) | 23 (13.3%) |
| Fizzy drinks | 39 (22.7%) | 93 (54.1%) | 40 (23.3%) |
| Alcohol | 44 (25.6%) | 49 (28.5%) | 79 (45.9%) |
| Water | 27 (15.2%) | 103 (57.9%) | 48 (27.0%) |
| **Physical Activity** |  |  |  |
| Moderate/strenuous activity | 83 (46.4%) | 49 (27.4%) | 46 (25.7%) |
| Walking activity | 43 (24.0%) | 60 (33.5%) | 76 (42.5%) |
| Sedentary time | 5 (2.8%) | 34 (19.0%) | 140 (78.2%) |
